# “It’s Not Secret—It’s Not Advertised” – Content, Format, and Platform Preferences to Promote PrEP Use in the Southern United States

**DOI:** 10.1007/s10461-026-05029-1

**Published:** 2026-01-19

**Authors:** Ronnie M. Gravett, Joseph D. Tucker, Lynn T. Matthews, Barbara Van Der Pol, Greer McCollum, Jason J. Ong, Jeanne Marrazzo, Latesha Elopre

**Affiliations:** 1 Division of Infectious Diseases, Department of Medicine, Heersink School of Medicine, University of Alabama at Birmingham, 1900 University Blvd, THT 225, Birmingham, AL 35294 USA; 2 Institute for Global Health and Infectious Diseases, University of North Carolina at Chapel Hill, Chapel Hill, NC USA; 3 Clinical Research Department, Faculty of Infectious and Tropical Diseases, London School of Hygiene & Tropical Medicine, London, UK; 4 Melbourne Sexual Health Clinic, Melbourne, Australia; 5 School of Translational Medicine, Monash University, Melbourne, Australia; 6 Birmingham, AL USA

**Keywords:** HIV Pre-exposure prophylaxis, Health promotion, Men who have sex with men, Qualitative research

## Abstract

Gay, bisexual, and other men who have sex with men (GBM) in the Southern United States (US) experience high HIV incidence yet have relatively lower HIV pre-exposure prophylaxis (PrEP) uptake, especially among GBM of color. Despite awareness, PrEP use remains insufficient to meaningfully impact the HIV epidemic in the Southern US. PrEP promotions largely focus on PrEP messaging, especially product awareness. Theory-based health promotion research has not explored how to develop promotions that drive PrEP uptake. To close this gap, we explored preferences among GBM in the Southern US for promotion content, format, and platform. Grounded in Andersen’s Model, we conducted semi-structured interviews with HIV-negative GBM, aged 18–39 years, and used inductive and deductive coding for thematic analysis to develop themes and sub-themes to understand promotion preferences. Forty GBM (68% Black, 10% Latino, 53% not using PrEP) completed interviews. Three major themes emerged: (1) content beyond promoting PrEP awareness, (2) using digital devices and media access for promoting PrEP, and (3) platforms for PrEP promotion. GBM in this study preferred digital promotion of PrEP, focusing on access, safety, and effectiveness that is delivered discreetly; promotion in the non-digital space could normalize PrEP use. Creating promotions that address these themes will make promotions more relevant to drive uptake of PrEP in the Southern US.

**Supplementary Information:**

The online version contains supplementary material available at 10.1007/s10461-026-05029-1.

## Introduction

 Gay, bisexual, and other men who have sex with men (GBM) comprise 70% of new HIV diagnoses in the United States (US), but slow HIV pre-exposure prophylaxis (PrEP) uptake hinders its population impact [1]. Although PrEP effectively prevents HIV acquisition, PrEP uptake among GBM in the Southern US trails behind the national trends, contributing to new HIV infections, especially among Black and Latino GBM, compounding the already disproportionately high rates of new HIV diagnoses in these communities; in the South, the PrEP-to-Need ratio (PrEP prescriptions to HIV diagnoses) among White persons approaches 20 (i.e., nearly 20 PrEP users per one new HIV diagnosis) compared to approximately 5 for Black persons [2]. In 2022, only 25.7% of Alabamians with need for PrEP received a prescription, leading to a modest declines in new HIV diagnoses (0.6% year-over-year) from 2012 to 2022 [3]. Despite the first US Federal Drug Administration approval of oral PrEP in 2012, slow PrEP uptake in the South has been attributed to structural barriers and low awareness [4]. While PrEP awareness is improving in the South, other factors such stigma, medical mistrust, and lack of access hinder successful PrEP uptake and scale up [5–7].

To improve PrEP utilization, efforts should focus on progressing through the continuum, i.e., progression from awareness to access to uptake and persistence, with a focus on shifting from mere awareness to actionable steps: accessing and engagement. PrEP promotional campaigns have successfully increased PrEP awareness in urban settings as determined by digital metrics and paradata, yet PrEP prescriptions have not increased among communities with highest need [8–10]. Moreover, recent data showed that rural persons had significantly lower PrEP awareness compared to urban persons (50% vs. 78%) after a national PrEP promotion campaign with most rural participants hearing about PrEP through TV, radio, or digital media rather than a healthcare provider or internet or social media source [11]. The behavior change at the awareness stage of the PrEP continuum is an underrecognized aspect for PrEP promotion, so understanding how to reach GBM with the necessary information to make this change from awareness to action is a key gap.

Health promotion for HIV testing and prevention services is often informed by behavioral theories of change, such as the health belief model, transtheoretical model of change, or theory of planned behavior; these theories focus on intrapersonal, interpersonal, and higher-level factors to promote behavior change, yet the role of the message to inform the behavior change is less clear [12]. Studies have demonstrated the role of message-framing for HIV prevention promotions, emphasizing gain-framing to demonstrate the benefits of PrEP, and acknowledging that the public perception of PrEP promotions may be stigmatizing [13, 14]. Although the promotional product (e.g., the flyer or the commercial video) serves as the vehicle to deliver the message, most research does not focus on understanding the promotional product’s role in leading to a behavior change, i.e., PrEP information seeking or engaging in PrEP care. For example, rural persons often note that stigma is a major barrier to accessing PrEP, so PrEP promotional messaging should feature affirmation and inclusivity to promote PrEP use [11]. Prior work demonstrated that GBM in the Southern US prefer PrEP promotions that feature inclusivity and diversity beyond just racial, sexual, or gender identities, foster relatability, and reduce the emphasis on HIV “risk,” yet little is known regarding how to craft the promotion itself [15]. In this study, we employed Andersen’s Healthcare Utilization Model to explore preferences for PrEP promotional content, formats, and platforms among GBM that would feature community-informed, inclusive, and affirming PrEP promotions to shift from PrEP awareness to engagement. Previously, we reported the preferred content in PrEP promotions, so this work focused on how GBM prefer to view this content [16].

## Methods

We explored the preferences for PrEP promotion among GBM in the Southern US by conducting 40 semi-structured interviews with GBM. Our inclusion criteria required (1) self-reported being aged 18–39 years old, (2) HIV-negative or unknown HIV status, and (3) English-speaking. We employed a phenomenological approach to this work to understand how lived experiences of GBM affect the way GBM prefer to receive PrEP promotions that could influence PrEP uptake [17]. We purposively sampled GBM in Birmingham, Alabama through social media channels, community outreach, and study participant registry with goals of at least 50% Black and 10% Hispanic/Latino, reflecting the demographics of local HIV diagnoses, and at least 50% participants not currently using PrEP [18].

**Fig. 1:**
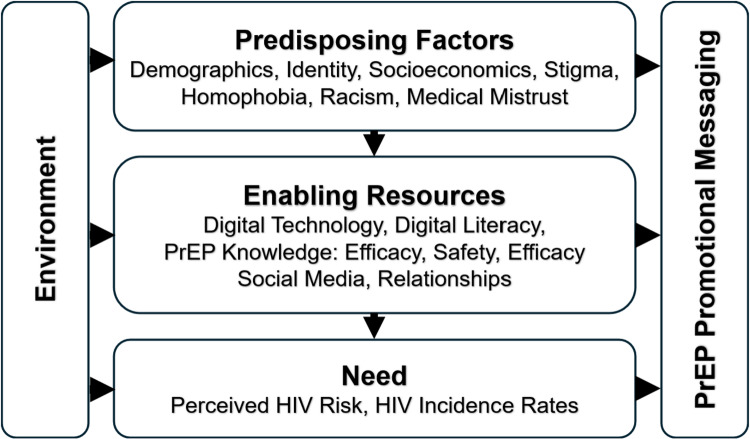
Interview topics grounded in the constructs of the Andersen’s Behavioral Model of Healthcare Utilization

We grounded the interview guide in the Andersen Behavioral Model for Healthcare Utilization (ABM) to develop topics mapping to ABM constructs: environment, predisposing factors, enabling resources, and need and their influence on PrEP awareness and use (Fig. 1) [19]. We iteratively revised the guide based on interviewer feedback and initial data review (see supplement for final version). Trained interviewers conducted one-on-one interviews either in-person or virtually via a secure video conferencing platform. Interviewers digitally audio recorded the interviews, and a secure, HIPAA-compliant transcription service transcribed each recording verbatim. We reviewed transcriptions for fidelity and completeness and uploaded them to NVivo qualitative data analysis software (Lumivero, Denver, CO, USA). Two coders deductively coded the transcripts based on a priori codes determined by literature review and ABM constructs and inductively coded to allow de novo codes to arise [20]. The first coder, with similar lived experiences to participants, contributed considerable reflexivity to better understand the context of these data [21]. Coders established the codebook, compared coding regularly, and iteratively revised the codebook until they produced the final codebook (see supplement). Using the final codebook, the two coders independently coded 25% of transcripts (10 transcripts) to measure intercoder reliability. Coders attained high intercoder agreement with a Cohen’s Kappa of 0.85 [22]. Using a thematic analysis approach, we operationalized codes into categories and then constructed them into themes with sub-themes. We considered how each theme and sub-theme best fit into the ABM constructs, and we linked each sub-theme back to ABM constructs; findings could be mapped to multiple constructs as appropriate (e.g., Environment and Enabling Resources).

## Results

Forty GBM completed one-on-one interviews from January 2023 to December 2023. Participants had a mean age of 28.2 years (SD 4.8 years). Participants identified their race as Black (*n* = 27, 68%), White (*n* = 11, 28%), or other race (*n* = 2, 5%), and 4 (10%) identified their ethnicity as Hispanic or Latino. Twenty-one (53%) reported not currently using PrEP, and 19 (47%) reported using PrEP at the time of the interview. The majority (*n* = 32, 80%) reported education beyond high school, and 26 (65%) reported income less than $50,000. Table 1 shows complete demographics.

**Table 1:** Sociodemographics and PrEP use characteristics of all participants

Sociodemographics	*N* = 40
Mean Age, Years (SD)	28.2 (4.8)
Race n (%)	
Black	27 (67.5%)
White	11 (27.5%)
Other	2 (5.0%)
Ethnicity n (%)	
Hispanic	4 (10%)
Non-Hispanic	36 (90%)
Highest Education n (%)	
High School Diploma or Equivalent	8 (20%)
Some College – did not graduate	6 (15%)
Associate degree	5 (12.5%)
Bachelor’s degree	14 (35%)
Graduate or Professional Degree	7 (17%)
PrEP Use n (%)	
Never	14 (35%)
Prior	7 (17.5%)
Current	19 (47.5%)
Income n (%)	
<$25,000	6 (15%)
$25,000-$49,999	20 (50%)
$50,000-$74,999	9 (22.5%)
>$75,000	3 (7.5%)
Prefer not to answer	2 (5%)

We found three major themes in this work: (1) content beyond promoting PrEP awareness, (2) using digital devices and media access for promoting PrEP, and (3) platforms for PrEP promotion. Table 2 shows themes, sub-themes, and exemplar quotes mapped to relevant ABM constructs.

**Table 2:** Themes, sub-themes, exemplar quotes mapped to constructs in andersen’s behavioral model for healthcare utilization

Theme	Sub-Theme	Exemplar Quote	Construct
Theme 1: Content beyond promoting PrEP awareness	Sub-Theme 1.1: Awareness may vary but generally felt to be good.	*Some people do know about PrEP and don’t take it…They just choose not to take it for some reason.* (28, Black, current PrEP user) *I feel like it’s becoming more and more known.* (24, White, former PrEP user) *I think they have an idea of what it is. But I think it’s really important to describe more about what it’s for.* (29, Black, never used PrEP)	Enabling Resources
	Sub-Theme 1.2: Focus on PrEP access, effectiveness, and safety	*I feel like there needs to be more of a documented list or something or a promoted list where it’s like*,* “Hey*,* you could get PrEP here. You can go here*,* within this area.” Like*,* “These are the people who are providing PrEP*,* whether you’re insured*,* you know—if you’re insured*,* you could go here. If you’re not insured*,* you can-you can go here.”* (23, Black, former PrEP user) *I definitely think [the PrEP promotion] should have information about it prevents HIV; it’s for everyone; it’s this percentage effective; it’s safe; maybe even having the QR code that can go to [redacted] or whatever agency is available*,* so you can check and see if it’s available in your city*,* state.* (34, Black, current PrEP user)	Enabling Resources
	Sub-Theme 1.3: Promotions should be concise, not overly detailed, and accessible.	*I like short and to-the-point commercials. I don’t like really drawn-out commercials…concise and clear.* (27, Black, never PrEP user) *Something that’s short*,* cause I know people attention span is very short. So something that people can look at and be like*,* “Oh*,* this is what you trying to say*,*” within five*,* ten seconds.* (26, Black, former PrEP user)	Enabling Resources
	Subtheme 1.4: Promotion content should avoid over sexualization	*I notice specifically a lot of the ads are kinda sexualized in a way. And I’m assuming that’s just to grab attention… Sometimes they kinda just rub me the wrong way ’cause I’m like*,* we’re more than that and more than just sexual beings*,* or that’s the main thing we care about.* (23, Black, former PrEP user) *Just a man by himself*,* and he would either be in a Speedo or somethin’…the ones where it was just the man by himself were more hypersexualized…I see you’re talkin’ about PrEP*,* but*,* you know*,* what does the half-naked man have to do with the value of PrEP?* (25, Black, never used PrEP) *I feel like that it should be promoted in a very general basis*,* like not a very sexually-driven ad or anything like that. I think that should be approachable ad*,* approachable promotion of it*,* I think would definitely be better in the community.*,* that way it’s easily accepted-accepted by all.* (38, White, current PrEP user)	Predisposing Factors
Theme 2: Using digital devices and media access to promote PrEP		*I am gonna be on my phone pretty much all day…I said I’m not really gonna watch commercials or listen to them*,* but if a [PrEP ad] comes across one of my sites that I’m on and I see it*,* I’ll be interested.* (27, Black, never used PrEP) *My phone I use mostly for connecting with friends and family*,* taking photos. Stuff like that. My phone is more personal and then my laptop*,* if I have to zone in on something I’m taking out my laptop.* (21, Hispanic, never used PrEP) *I use [digital devices] for like everything. I mean I play games on them. I use the dating apps. I read my news on*,* on it. I use ’em for everything…I mean it’s where I see the most PrEP ads at*,* and it’s where I notice them at.* (37, former PrEP user)	Enabling Resources
Theme 3: Platforms for PrEP promotion	Sub-Theme 3.1: Physical signage puts PrEP in the present, physical space to normalize its use.	*A billboard probably be very good. Something like a billboard. (*23, Black, never used PrEP*)* *I also think just in public view*,* like on billboards*,* or city buses. I think that can be appropriate and advantageous too.* *(*32, White, current PrEP user) *I travel so much. When I go to Atlanta*,* it’s everywhere. Like*,* you can go downtown. They have a billboard up for PrEP. I haven’t seen a billboard up with PrEP down here yet… they’re not advertising the way they need to be advertised.* (24, Black, never used PrEP)	Environment
	Sub-Theme 3.2: Static graphics and videos are both adequate media for promotion	*I feel like nowadays*,* because people are so used to short videos and stuff*,* actually*,* just a picture with the important information would be better… a simple post will be more effective* (33, Asian, current PrEP user) *Videos would probably be more effective than just a regular ad…TV and videos definitely probably are the top priorities or ways you can probably get that message out to people.* (23, Black, never used PrEP) *I get annoyed when I’m trying to talk to somebody*,* and then I have to sit through a ten-second video. I’d rather just a graphic show*,* come up…the way the apps work with Grindr*,* and Scruff work is like you’ll talk to somebody. And*,* when you push the back button*,* to go to your list of conversation*,* they serve you an ad.* (37, White, former PrEP user)	Enabling Resources
	Sub-Theme 3.3: Venue-based and in-person promotion were commonly suggested to promote PrEP	*I think it would be cool to see it advertised at drag shows.* (31, White, current PrEP user) *Different live events such as like drag shows and bars*,* and of course medical places.* (27, White, former PrEP user) *Let’s say for instance*,* uh*,* during Pride*,* I think they normally set up a little station that has information with fliers and stuff like that. And I think that’s important too… And it’s also gives the in-person aspect of that. And if somebody had a question at that time*,* they’re able to ask them. I think that’s a good thing.* (35, White, never used PrEP) *I think education communities*,* universities*,* high schools*,* um*,* maybe community like events*,* or community athletic programs. certainly at community or public health institutions.* (32, White, current PrEP user)	Environment Enabling Resources

### Theme 1: Content Beyond Promoting PrEP Awareness

Participants had varied opinions as to whether persons who need PrEP were aware of PrEP, although many participants felt that most GBM who would benefit from PrEP were already aware of PrEP. As such, several participants noted that messaging should be more salient, or relevant to appeal viewers to act on using PrEP.

#### Sub-theme 1.1: Awareness May Vary but Generally Felt to be Good

Although most participants felt that there was high PrEP awareness, participants felt that several persons who could benefit from PrEP were unaware. One participant noted, “*there’s a huge amount of people who do not know that they would be great candidates for PrEP.”* (32, White, current PrEP user). Another participant described how awareness may be incomplete or insufficient, “*I would say yes and no because I do feel like there are a lot of people who know about PrEP…but don’t know many details about it.”* (26, Black, current PrEP user). While GBM might be aware that PrEP exists, they do not know more details about the modalities, efficacy, safety, or access.

Furthermore, participants felt that PrEP awareness was insufficient to lead to behavior change, and that understanding how PrEP works and key steps to access it would make persons more likely to use PrEP. Providing relevant information about PrEP could potentially help persuade persons to start using PrEP. One participant stated, “*I wouldn’t wanna take something that I don’t know anything about*,* or you just telling me to take it because it’ll prevent it. But what is it? Are there side effects? What’s gonna happen if I take it? Is it for me?”* (27, Black, never used PrEP). This participant introduced the notion that more information about PrEP would help with self-efficacy and decisions about using PrEP. Another participant furthered this idea to also include a notion of medical mistrust, “*I’m not gonna believe in giving folks something*,* having folks take something*,* and they have no knowledge of what they’re taking.”* (28, Black, Current PrEP user).

#### Sub-theme 1.2: Focus on PrEP Access, Effectiveness, and Safety

Most participants discussed that PrEP promotions should prioritize how to access PrEP, focusing on cost and location, as well as effectiveness and safety. They felt that including practical information about how to access PrEP would overcome some structural barriers to care, as one participant stated,

“*I’ve never seen an ad discuss where to get it. It just says ‘call your doctor*,*’ and again 65% don’t even have a primary care doctor…ads should explain where to get it from*,* how to get it*,* different ways to get it*,* programs*,* or whatever…but it’s not advertised. It’s like it’s secret. Like everything—it’s not secret…it’s not advertised for people to actually go get help.”* (24, Black, never used PrEP).

This participant highlighted key issues with accessing PrEP beyond just unawareness but also noting lack of healthcare access with perceived low primary care availability. The tone of this statement intimates a feeling of frustration with GBM who desire PrEP but are unable to access it. Participants reported that specific information, e.g., “different ways to get it, programs, or whatever” would be helpful to guide the promotion’s audience towards the next steps in using PrEP. Participants also noted the importance of mentioning safety and efficacy in addition to accessibility, as one participant remarked,

*“It would be definitely like accessibility at the top. Like accessibility is huge for it ‘cause I know that it is not as accessible as it should be. it’s definitely those three things…So safety*,* efficacy*,* and accessibility. Hands down three most important things.”* (32, White, current PrEP user).

#### Sub-theme 1.3: Promotion Messages Should be Concise, Not Overly Detailed, and Accessible

Participants felt that too many details in the promotions would be overwhelming for some viewers. Furthermore, participants felt that many details could be saved to discuss with a PrEP provider. Additionally, participants felt that GBM would pay closer attention to more concise promotions, and, in some instances, GBM would skip or ignore longer promotions. One participant stated, “*It’s just so much that it’s just like*,* okay*,* I’m not interested. So*,* I feel like the less information*,* but the most important information is effective…I wouldn’t look at an ad if it had a lot of just words and stuff.*” (32, Black, current PrEP user).

To be approachable and accessible to the GBM community, participants described successful promotions as employing an interpersonal or peer-level approach rather than describing PrEP through a clinical or public health lens. They noted using less clinical or medical jargon and utilizing anecdotes to discuss PrEP, as one participant stated, “*kind of not with the big words and stuff*,* just talking to us at a level like our friends do.”* (26, Black, former PrEP user). Accessible language, and more approachable promotions, would also help normalize PrEP.

#### Sub-theme 1.4: Promotion Content Should Avoid Over Sexualization

Many participants felt that messages often featured excessive sexual meanings or conveyed a sexual tone, which was not desirable to these participants. Participants described these scenarios as promotions featuring men with little covering or clothing, suggestive or explicit language, or models in overtly sexual situations, such as in bed or taking suggestive pictures. Participants did not feel like such promotions were motivating; they felt that such images of men re-emphasized that PrEP is for gay men only instead of for everyone. Moreover, they felt that having promotions featuring a less sexual nature or tone would normalize PrEP and make PrEP seem more accessible to larger communities with need for HIV prevention. One participant described this, “*I notice specifically a lot of the ads are kinda sexualized in a way…sometimes*,* they kind just rub me the wrong way ‘cause we’re more than just sexual beings or like that’s the main thing we care about.”* (23, Black, former PrEP user). Another participant stated, “*I see you’re talking about PrEP*,* but what does the half-naked man have to do with the value of PrEP.*” (25, Black, never used PrEP).

### Theme 2: Using Digital Devices and Media Access to Promote PrEP Messages

Participants reported frequently using digital devices throughout all aspects of their lives and daily routines. Some participants noted a distinction in which devices they used for different tasks. Most participants remarked that nearly all PrEP promotions that they viewed or experienced were through a smart digital device, i.e., cell phone, tablet, or computer, and few participants recalled PrEP promotion in their digital space.

All participants reported frequently accessing social media through their personal digital device. Participants each reported their preferred social media, and why they accessed those media apps or websites. Participants noted the importance of considering age and other demographic aspects when selecting a social media platform to promote PrEP. While not all participants reported actively using dating apps or sex-finding apps at the time of the interviews, all participants were familiar with these types of apps, often mentioning Grindr, Jack’d, and Scruff as common apps. Those that had used these apps reported seeing PrEP promotions, either as a banner ad, a full-screen ad, or even a video promotion. Additionally, few participants also reported experience using gender-neutral apps, such as Tinder, but they did not recall interacting with PrEP ads on that platform. Participants reported differing engagements with attention to the ads with only a few participants noting that the promotions interfered with their intended use of the app.

### Theme 3: Platforms for PrEP Promotion Messaging

Participants reported varied perspectives on how to promote PrEP. The most consistently discussed topics included aspects of digital promotion via social media, sex-finding apps, video streaming platforms, and websites. However, several participants also discussed the value of promoting PrEP in the physical space, through posters and signage, including billboards and flyers, and through in-person outreach using key influencers, such as drag performers. Importantly, the notion of promotion in a physical space made PrEP seem more “real world,” current, and tangible, again suggesting the importance of normalizing PrEP use.

#### Sub-theme 3.1: Physical Signage Puts PrEP in the Present, Physical Space to Normalize Its Use

Participants often mentioned billboards, posters, and other physical signage as important ways to promote PrEP, and participants considered these strategies as relatively low intensity, passive promotion and should be more widespread. Billboards and posters could make PrEP seem more tangible and real-world than exclusively digital promotion. One participant stated, “*I haven’t seen a billboard up with PrEP down here yet…they’re not advertising the way the need to be advertised*” (24, Black, never used PrEP).

#### Sub-theme 3.2: Static Graphics and Videos are both Adequate Media for Promotion

Static images can be easily shared on social media or other digital media platforms and posted or distributed in the physical space. Some participants felt that static graphic would be preferred as viewers would be less likely to skip them as they would skip videos. Static graphics would also be less restricted as to where or how they can be used. Videos were also commonly mentioned as ways to promote PrEP. Participants often described video promotions as commercials or promotions preceding or during videos on a streaming service. Participants most often described these videos being shared via video social media platforms, such as Instagram or TikTok. Other participants described frustration with video promotions as they often lasted too long or interfered with their main focus to use the app or watch the video, which made them feel less responsive to the promotion as they skipped video as soon as possible.

#### Sub-theme 3.3: Venue-based and In-Person Promotion Were Commonly Suggested to Promote PrEP

Participants reported that promotion through venues would be an effective way to reach audiences who need to know about PrEP. Common venues mentioned included social spaces, such as bars or clubs. Participants frequently felt that outreach-based promotions would be helpful to promote PrEP. Leaning into interpersonal communication as a way of promoting PrEP, participants mentioned that it would be helpful just to have PrEP promoters visible in venues or at events.

Additionally, many participants felt that schools would be an excellent way to reach young persons (adolescents and young adults) who might be at risk for HIV but not understand their risk for HIV. As such, participants frequently described “campus” as an ideal location, implying a role for higher learning institutions, i.e., colleges or universities. However, other participants also recognized that discussions related to PrEP or HIV, especially in the context of LGBTQ + identities, may not be allowable in other educational venues, even though there would be a need.

## Discussion

This work provides the first, in-depth understanding of how GBM prefer PrEP promotions that they believe would be more effective to increase PrEP use in the unique, Deep South context. Furthermore, this work extends our understanding of PrEP promotion by describing the preferred formats and platforms to deliver the promotional message. The study sample represented a diverse GBM cohort with varied racial, ethnic, educational, income, and PrEP use backgrounds, reflective of the Southern United States epidemiology. Importantly, the research team’s lived experience served to increase the reflexivity on this work, allowing a richer, deeper understanding of these data.

Participants emphasized preferences for digital PrEP promotion as well as promotions beyond the digital realm, considering the physical space to help normalize PrEP promotion and increase the promotion salience. This study emphasized how the digital space, especially social media and dating apps, conveniently and discreetly allows PrEP information to be at viewers’ fingertips, which is critical for dispersing useful information. Recent studies demonstrated that PrEP campaigns may be framed as private or public, but PrEP users and GBM comfortable with disclosure preferred public or neutral framing [23]. Our data show that GBM recognize and prefer the convenience and discretion of digital messaging, but public and venue-based PrEP promotion would also normalize PrEP use and also improve the salience of the promotion to viewers. Furthermore, our data shows that GBM regularly access digital platforms and use digital media. To do so, this requires digital device access, which is not always reliable, especially in communities that are highly affected by poverty, housing instability, and low digital literacy rates, which are overrepresented among marginalized communities [24]. As such, PrEP promotions should continue to have presence in non-exclusively digital spaces (e.g., billboards, signage, radio, print) to ensure a broader reach. PrEP promotions in the physical space normalizes PrEP use, which would increase its broader appeal to all communities and help reduce stigma driven by targeted advertisements, such as promotions featuring only LGBTQ + community members or only persons of color [25]. Using institutional venues, such as schools or college campuses, and social venues, such as bars or cafes, to promote PrEP could also help normalize PrEP. Considering the Environment construct of the ABM, we determined that both the digital and physical environments featuring these promotions directly impact other aspects of the PrEP promotion as enabling resources or predisposing factors, e.g., promotion in the digital space requires digital technology access and literacy. These data should implore public health authorities, community organizations, and PrEP providers to diversify their promotion portfolio to not solely rely on digital messaging as a means for conveying helpful information.

GBM in this study voiced preferences for concise, practical, and actionable promotional content to help the promotion audience or viewers know or learn how to access PrEP services effectively. Creating PrEP promotions that meet these criteria can serve as enabling resources to shift PrEP promotion to actionable steps to start using PrEP. Indeed, many social media campaigns focused on describing or defining PrEP, which successfully improved PrEP awareness, although unable to prove increased uptake [8, 9, 26]. Participants emphasized their desire to know about cost and location of PrEP services to streamline their ability to start PrEP, facilitating the behavior change from awareness to action. While understanding the efficacy and safety of PrEP remains important, these topics do not mutually exclude more details about access, such as PrEP providers and assistance programs. Shifting the focus of the messaging to action messages will allow viewers of the promotion to more clearly see the next steps to access PrEP and facilitate the behavior change needed to improve PrEP uptake and reduce disparities. Promotion messages that provide details about efficacy, safety, and access operate as an enabling resource to move beyond PrEP awareness to PrEP use.

Sex-positive promotions succeeded in improving PrEP awareness in some areas, particularly coastal cities and urban centers [27]. These promotions, featuring models in intimate poses and risk-reducing, evocative language, captured the intended audience’s attention to increase awareness [9]. Our work demonstrated that many GBM in the Deep South are less receptive to promotions they perceive as “hypersexual,” in part due to desire to normalize PrEP use away from sexual messaging, and the sexual and HIV stigma, more common in the Southern US, likely also contributes to this preference [28, 29]. This aligns with other work demonstrating PrEP users’ comfort with PrEP framed as a public or neutral matter rather than private. To reach GBM not using PrEP or not comfortable with disclosure, overtly sexual promotions may not be successful, especially in a public frame [23]. Sex-positive promotions, which may be empowering in some settings, could be weaponized through undue scrutiny, stigma, homophobia, and racism in Southern US settings, so efforts to normalize PrEP inclusive and affirming promotions, away from sexualization, will overcome predisposing factors such as stigma and medical mistrust.

Participants frequently mentioned the importance of social media in promoting PrEP, yet social media influencers may be underutilized as a way to promote PrEP and other prevention tools [30]. Recent data demonstrate that content with Black, gay social media influencers lead to increased PrEP interest among Black, gay social media users when compared to paid advertisements [31]. Social media influencers foster relatability in a promotion, i.e., how a viewer can relate to the person in the promotion, a key promotional strategy to increase promotion relevance. Our data support the concept that social media influencers could lead to improved digital engagement based on relevance and relatability. This supports prior work showing that GBM preferred relatability often delivered from a personal connection or community leader [15].

PrEP promotions have increased PrEP awareness in high incidence, urban areas, but it is difficult to know how this translates into access and engagement for PrEP uptake [2, 13]. One challenge in implementing PrEP promotional campaigns is how to evaluate the outcomes and the success of the campaign. While evaluators can measure digital metrics, such as website or mobile paradata, (e.g., QR code access, website views, click through ratios), the challenge remains in linking the promotion impact to the behavior change [14]. Designers, implementers, and evaluators of future PrEP promotional campaigns should consider this critical aspect of evaluation since demonstrating effectiveness will direct public health resource allocation.

We recruited some participants in this study via digital platforms, which could contribute to sampling bias; however, nearly all participants ubiquitously mentioned the significant role of digital access, even if not recruited through digital sampling. Additionally, this study centers GBM from the Deep South, which limits generalizability to other regions, but the southern region is disproportionately affected by the HIV epidemic and warrants focused attention to the unique context of PrEP promotion in this region among a diverse population of various racial, ethnic, financial, and educational backgrounds.

In conclusion, GBM in this study preferred digital promotion of PrEP, focusing on access, safety, and effectiveness, yet promotion in the non-digital, physical space should not be abandoned, especially in efforts to normalize PrEP use. Understanding these aspects should influence tailored PrEP promotional campaigns and behavioral interventions that move from PrEP awareness to improved PrEP access and uptake.

## Supplementary Information

Below is the link to the electronic supplementary material.

Supplementary Material 1

## Data Availability

Participants did not provide explicit consent to share their data, and, as this study collected potentially sensitive data, the consent process does not allow data sharing.
