# Supplementary material for: “It’s Not Secret—It’s Not Advertised” – Content, Format, and Platform Preferences to Promote PrEP Use in the Southern United States
Source: AIDS Behav. 2026 Jan 19;30(7):2043–52. doi: 10.1007/s10461-026-05029-1 (PMC13400677; doi:10.1007/s10461-026-05029-1)
Supplement: Supplementary file 1 — Supplementary Material 1 [file 10461_2026_5029_MOESM1_ESM.pdf]

Supplementary Materials

AIDS and Behavior

“It’s not secret—it’s not advertised” – Content, Format, and Platform Preferences to Promote PrEP Use in the Southern United States

Ronnie M. Gravett, MD, MSPH[§], Joseph D. Tucker, MD, PhD, Lynn T. Matthews, MD, MPH, Barbara Van Der Pol, PhD, MPH, Greer McCollum, MPH, Jason J. Ong, MBBS, PhD, Jeanne Marrazzo, MD, MPH, and Latesha Elopre, MD, MSPH

§ Corresponding Author:
Ronnie M. Gravett, MD, MSPH
rgravett@uabmc.edu

Supplement 1: Semi-structured interview topic guide

Supplement 2: Final codebook

Interview Guide
(60-90 minutes)

Study Title: Amplifying Our Voice: Understanding HIV PrEP Promotional Messaging to Create Authentic PrEP Content by Men Who Have Sex with Men in the Deep South

IRB Protocol Number: IRB-300008162

Principal Investigator: Ronnie M. Gravett, MD

Sponsor: National Institute of Mental Health/National Institutes of Health

Participant Study ID: __________

Date of Interview: __________

Time Started: __________

Time Ended: __________

Informed Consent Process:
- Review the Informed Consent Form with the Participant
- Answer any questions for the Participant
- If requested, the Participant can review the Informed Consent Form for 24 hours
- The Participant will sign and date the Informed Consent Form, if in-person. If the interview is conducted virtually, then the participant will give verbal consent to participate.
- The Interviewer will sign and date the Informed Consent Form. If the interview is conducted virtually, then the Interviewer will indicate that verbal consent was given.

**Table 1. Topics Grounded in the Andersen Behavioral Model**	
**Predisposing Factors**	Demographics, Sexual Behaviors, Homophobia, Stigma, Medical Mistrust,
**Enabling Resources**	Income, Education, Enabling Relationships, Community Engagement, Disclosure Status, PrEP Knowledge, Digital Access
**Need**	Perceived need, objective need

Introduction:

Thank you for taking the time to talk with me today about this topic. You have been invited to participate because you have reported that you identify as a cisgender man who has sex with other cisgender men or transgender persons.

We are interested in understanding more about how gay, bisexual, same gender-loving, and other men who have sex with men feel about HIV pre-exposure prophylaxis (PrEP) promotional messaging and content. PrEP is very effective at preventing HIV among persons at-risk, but little is known about how to effectively promote PrEP to the communities that need it most. We will ask questions about your feelings regarding PrEP and its effectiveness, how you perceive its promotion, and what aspects or factors you believe are important to promote PrEP. We will also ask you some questions about yourself. Your experiences are important, so please feel free to speak openly and honestly.

This interview will be audio recorded to ensure accurate representation of your thoughts. We will not collect nor store any personal information along with your responses. To help us ensure confidentiality, we ask that you do not use names of yourself or others during this recording. We will keep all information, including the recordings, private in a secure location at UAB with access limited only to approved study staff.

You may stop at any time during this conversation. Please let us know if you need a break. Before we start, are there any questions now?

<u>Demographic and Socioeconomic Survey</u>

How old are you right now?

- _______

How do you identify your race?

- American Indian or Alaskan Native
- Asian
- Black / African American
- Native Hawaiian or Pacific Islander
- White
- Multiple Races (specify ________________________)
- Other (specify: ______________________________)

How do you identify your ethnicity?

- Hispanic / Latino
- Not Hispanic / Latino

What is the highest level of education that you have completed?

- Did not graduate Highschool
- Highschool Diploma or equivalent
- Some college, did not graduate
- Trade or Vocational Training
- Associates Degree

- Bachelor's Degree
- Graduate or Professional Degree

What is your approximate annual income?

- < $25,000
- $25,000 - $50,000
- $50,000 - $75,000
- > $75,000
- Prefer not to answer

Do you currently use PrEP?

- Yes
- No

Have you ever used PrEP in the past?

- Yes
- No

<u>Interview Questions:</u>

Icebreakers

Tell me about what kind of media do you access.

Probe: Do you watch TV, movies?
Probe: Do you use the internet? How?
Probe: Do you use social media or certain other apps?

Tell me about any kind of PrEP advertisement or promotional messages you've seen.

Probe: If you've seen a PrEP ad, how would you describe the ads?
Probe: Where did you see them? Who or what was in the ad?
Probe: How did they make you feel? How did you respond to them?
Probe: Do you feel like you would be responsive to them? What about others you know?

If you could describe a "perfect" PrEP ad that would encourage people like you to explore the idea of using PrEP or get more information about PrEP, what would that look like?

Probe: What would an ad that speaks to you or motivates you look like?
Probe: Where would you see it? Who would be in it?
Probe: What information or message would be included?

What formats of PrEP promotional messaging would be most effective?

Probe: Do you think graphics or videos would be more effective?
Probe: Where would you prefer to see these messages or ads? (e.g., TV, radio, social networking apps, websites)

Predisposing Factors

Some people have experiences that might affect their ability to use PrEP or even know about PrEP. Your lived experiences are important to help us understand how to better promote PrEP.

The next several questions will ask about your lived experiences and how you perceive PrEP promotional messaging through those experiences.

How important is it to you to see persons in PrEP promotional ads that you can identify with?

Probe: Do you feel it is important to see a person who you feel has a similar identity as you?

Probe: What parts of that identity are important to see in PrEP promotions?

Probe: What parts are less important?

Can you tell me about how a person's race and/or ethnicity could influence feelings about a PrEP promotional ad?

Probe: How do you feel about seeing persons of the same race or ethnicity as you in PrEP promotion?

Probe: Would you feel that seeing persons of your same race in a PrEP would be stigmatizing?

How do you feel about PrEP promotional messaging that has persons with similar sexual orientation or behaviors?

Probe: How do you feel about seeing other Gay, Bisexual, Same-Gender Loving, or other men who have sex with men in PrEP promotions?

Can you tell me how you feel about your sexual orientation or sexual behavior these days?

Probe: Have you ever experienced stigma or shame related to your sexual orientation or sexual behaviors?

Probe: Have you ever experienced this from a healthcare provider?

Probe: Have you experienced positive feelings about your sexual orientation or behaviors?

How have your feelings around your sexual orientation or sexual behaviors influenced you using or not using PrEP?

Probe: Can you explain any kind of stigma or shame you felt about PrEP?

Probe: Do you think that stigma or shame influences others in your community about using PrEP?

Probe: What about any positive feelings about your sexual orientation or sexual behaviors influencing you using or not using PrEP?

Have you ever experienced any feelings or emotions related to PrEP promotional messaging?

Probe: Do you ever experience feelings of homophobia or homonegativity when viewing or PrEP messaging? If so, in what way?

Probe: Have you ever experienced feelings of stigma or shame related to PrEP? If so, in what ways?

Probe: Have you experienced any positive feelings related to PrEP promotional messaging?

How do you feel about PrEP promotional messaging discussing shame or stigma related to sexual orientation or sexual behaviors?

Probe: How so? Can you explain further?

How do you feel about trusting PrEP promotional messaging from healthcare providers, such as doctors, nurses, or clinics?

Probe: How do you feel about trusting PrEP promotional messaging from health authorities, like the health department or the CDC?

Enabling Factors

Some aspects of PrEP promotional messaging are important to help persons find or use PrEP, such as knowing about the medication itself or how to access the medication.

Do you feel that PrEP promotional messaging should prioritize describing or informing the viewer about what PrEP is?

Probe: Do you think that people who need PrEP know about PrEP?

<u>The medication</u>

Do you feel that PrEP promotional messaging should mention effectiveness in preventing HIV?

Probe: How important is the effectiveness when promoting PrEP?

Probe: Do you think many people may or may not know about PrEP effectiveness?

Do you feel that PrEP promotional messaging should mention its safety?

Probe: How important is the safety of PrEP when promoting it?

Probe: Do you think many people may or may not know about PrEP safety?

<u>Access</u>

How accessible do you feel PrEP is in your community?

Probe: What do you think are some issues that make PrEP more difficult to get?

Probe: What do you think are some aspects that make PrEP easier to get?

How do you feel about promotional messaging discussing where to get PrEP?

Probe: Do you feel that finding a PrEP provider should be promoted?

How do you feel about promotional messaging discussing PrEP costs?

Probe: Do you feel that PrEP costs should be included in the promotional messaging? Why or why not?

If you had to choose, what do you feel are the most important aspects about PrEP that should be promoted? Which aspects of PrEP are less important?

Probe: Can you explain what aspects are more important than others or why some may be less important?

<u>Enabling relationships</u>

Do you personally know anyone who takes PrEP?

Probe: Do you know of any friend who take PrEP?

Probe: Do you know of any sex partners who take PrEP?

Probe: How do you feel about discussing PrEP with sex partners?

Probe: How do you feel about discussing PrEP with other persons close to you?

How would you feel about someone you personally know delivering PrEP promotional messaging?

Probe: How would you prefer to receive such messaging from someone you know?

There are many ways to share information, such as social media, dating or sex-finding apps, print materials, etc. How would you like to hear about PrEP from someone you know?

Probe: Do you feel that you would rather receive PrEP promotional messages

How would you feel about PrEP promotional messages or ads showing friends, partners, or family members, etc.?

Probe: Would you feel differently if it showed friends or partners as compared to family members?

<u>Community Relationships</u>

Communities are an important part of our lives. People often belong to multiple communities that may be very influential in your life. Communities may be where you live, but communities can also be based on your identities, such as being a member of LGBTQ+ community, your religious or spiritual beliefs, your race or ethnicity.

How do you feel about community members, particularly community leaders, promoting PrEP?

Probe: Which communities do you think would be best to promote PrEP?

Probe: Would you feel community members or community leaders would be effective at promoting PrEP? If so, how could they be effective?

How do you think PrEP should be promoted within the community?

How do you feel about community organizations promoting PrEP?

Probe: Do you think community organization promoting PrEP is effective?

Probe: How trustworthy do you believe community organizations would be to promote PrEP?

What kind of community organizations do you think would be most effective?

Probe: How do you feel about religious organizations or communities promoting PrEP?

Probe: How do you feel about LGBTQ+ organizations promoting PrEP?

Probe: How do you feel about school or professional organizations promoting PrEP?

<u>Digital Access and Use</u>

Some people use digital devices such as smart phones, tablets, or computers to access to access all kinds of information. Some persons may use them to meet people or learn information about themselves, including health information.

How do you use digital devices?

Probe: Do you use digital devices to meet people for relationships or for sex? If so, how?

Probe: Do you use websites or phone apps to meet people?

Probe: Do you use digital devices to learn more about HIV, how to prevent HIV, PrEP, or other sex-related questions? If so, how?

How would you feel about digital PrEP promotion?

Probe: How effective do you feel using digital promotion for PrEP through websites or apps would be? Why or why not?

Probe: Which digital platform do you think would be effective? Which would not be effective?

Need

Do you feel that you are at risk for getting HIV?

Probe: Why or why not?

How do you feel about PrEP promotional messaging discussing risks for HIV?

Probe: What risks do you feel should be discussed?

Probe: Should PrEP promotion mention rates or how common HIV infection is? Why or Why not?

Probe: Should PrEP promotion mention sexual behaviors that increase chances for acquiring HIV? Why or why not?

How effective would it be for PrEP promotional materials to describe which persons may be at risk for HIV?

Probe: How would you feel about PrEP promotions describing certain persons or communities as having a higher chance for HIV?

Probe: Do you feel PrEP promotions mentioning certain persons, groups, or communities would be stigmatizing?

Probe: Should PrEP ads describe who is at risk for HIV?

<u>**Conclusion**</u>:

Thank you for talking with me today. I have no further questions at this time. Is there anything else that anyone would like to add?

On behalf of our study staff, we really appreciate your time to discuss this topic with us. Your insights are invaluable and will help us to better promote PrEP and other sexual health services to the community. The study coordinator will be in touch to ensure that you receive the compensation for your time and participation.

Amplifying Our Voice

Codes

Name	Description
ENABLING FACTORS	
Community Relationships	These are the relationships persons have with the community that they identify as being a part of. Any aspect of belonging to a community.
Community - leaders	Persons, or relationships with persons, who lead various communities. This could be religious leaders, communities advocates, community champions.
Community - organizations	The organizations, regardless of how formally assembled, that represent the “body” of the community
Community Mentoring	Mentoring or coaching programs within the community, may be formal or informal
Other Community	Any mention of different types of communities that can be based on collective similar identity, religious, or other cultural similarities (e.g., music, food, etc.) These may or may not be specifically linked to an organization but is not about the organization (e.g., LGBTQ community but not affiliated with LGBTQ organization like Magic City or TAKE).
Digital Access	Access to the digital world, particularly focusing on tools/devices to access and how someone accesses digital content
Cellphone	Subcode for using cellphone or smart phone

Name	Description
Computer	Subcode for using a computer
Digital Device - frequently use	Any mention related to frequently using a digital device
Other Digital Device	Subcode of any non-phone, non-computer, and non-tablet digital device for accessing the digital world (e.g., gaming device)
Tablet	Subcode for using a tablet (non-computer)
Enabling Relationships	Personal and Inter-personal level relationships with various persons. May include small group relationships (friend groups, group chats), but a level below the community level. These relationships may be family members, friends, colleagues or peers, sex partners, etc.
Family	Subcode for family relationships or aspects related therein.
Friends	Subcode for Friends or close personal contacts that are not necessarily sexual (although not restricted)
Personally delivered promotion	Any idea or mention referring to an inter-personal promotion of PrEP from one person to another via an established relationship (regardless of type)
Sex Partners	Subcode for any idea or mention about PrEP promotion or discussion among or between sex partners
Media Access	Access to media, including the type of digital or non-digital content that is accessed
Games or Gaming Apps	Accessing media to play games, using apps that are games, or any aspect of gaming
Internet as a tool	Using the internet for research

Name	Description
Media Use Frequency	Any mention of how often media is accessed/used
News or Newspaper	Any mention of news, including digital news or print news
Sex-finding Dating Apps	Geospatial dating apps or websites. This includes web-based platforms (websites) and app-based platforms (e.g., Grindr, Jack'd, etc.)
Social Media	Accessing social media via apps or website
Facebook	Subcode for Facebook
Instagram	Subcode for Instagram
Snapchat	Subcode for Snapchat
TikTok	Subcode for TikTok
Twitter	Subcode for Twitter
Streaming Services	TV or movie streaming services, which can be apps or websites
YouTube	Subcode for YouTube
Websites	Any websites that are not other categorized as news, sex-finding, or social media.
Positive Emotion	A "good feeling" related to PrEP, including its personal use, its discussion, or any other aspect of PrEP that causes a positive emotion.
PrEP Access	Any mention of how to access PrEP or PrEP services

Name	Description
PrEP Access - cost	Specifically referencing PrEP cost as related to accessing PrEP
PrEP Access - location	Specifically referencing the location of PrEP services or where to find PrEP services, as related to accessing PrEP
PrEP Awareness	Text that highlights whether people are aware that PrEP exists and that is for HIV prevention, not for text about PrEP specifics that would likely fall under the code "PrEP Knowledge."
PrEP Benefits	Any mention of non-specific PrEP benefits without more specifically mentioning effectiveness, safety, etc.
PrEP Effectiveness	Any mention of how effective PrEP is at preventing or stopping HIV acquisition.
Protection	Any mention of PrEP effectiveness as being “protective”
PrEP Knowledge	Any mention of knowing how PrEP works, effectiveness, safety, etc.
Misunderstand PrEP	Subcode for misunderstanding any aspect of PrEP care
PrEP Options	Code for mentioning different modalities for PrEP, i.e., oral pills, long-acting injection, etc.
Safety	Mentioning the safety of using PrEP
NEED	
HIV Risk	Any mention about the risk for HIV in general, including HIV incidence/prevalence or any mention of the notion of “how much” HIV there may be in a community, region, state, etc.

Name	Description
Persons at-risk	Any mention about HIV risk and whether a person is at risk based of aspects of their identity that may affect that risk.
Sex Risk Behaviors	Mentioning certain sex acts or behaviors as to whether or not increases risk for HIV
PREDISPOSING FACTORS	
Identity - Category	
Age	
Appearance and Presentation	Code for how a person presents themselves more generally, including body type, but not inclusive or racial or ethnic identities or other innate aspects of identity
“everyday people”	In vivo code for appearance of persons does not seem stylized
Diversity	Mentioning about multiple identities presented together, i.e., multiple race or ethnicity persons, multiple sexual or gender identities, religious, or cultural factors, etc.
“For Everyone”	In vivo code for the idea or concept that PrEP should not be limited to any particular identity, group, or community
Promotion Gender	The gender identity of the persons included in the promotion or ad or the person presenting the ad. Inclusive of cis-, trans-, NB/NC, and other gender identities.
Racial and Ethnic Identity	Mentioning the race and/or ethnicity of the person in the promotion/ad or the person presenting the promotion/ad
Colorism	Subcode for diversity based on skin tone among POC

Name	Description
Self-Identify	Any mention of “seeing yourself” or any aspect of a person’s own identity, including similar lived experiences, represented in a promotion or ad. Relatability is a key feature of this.
Sexual Orientation Identity	Mentioning aspects related to sexual identity or adjacent to this, i.e., heterosexual, homosexual, bisexual, etc. Would also include anything about “gay” or “straight.” This does not include gender identity.
Ignore Ads	Any text mentioning persons who may or may not be on PrEP (or potentially know about PrEP) but will ignore ads because of already knowing about PrEP or not being interested in PrEP at that time
Medical Trust	Mentioning how someone may trust or distrust the medical or healthcare establishment, including research.
Health Authority Trust	Subcode for medical trust specifically involving health authorities including public health agencies at any level, research institutions at any level, and Pharma or other industry organizations. This does not involve providers or local organizations
Provider Trust	Subcode of medical trust to more specifically identify trust or mis-/distrust from providers (MD, NP, PA, RN, etc.) that are individuals, small group practices, or affiliated with organizations. This would also include small community organizations. This does not include any health authority institutions.
Shame or Stigma	Experienced or internalized stigma or shame
PrEP Shame or Stigma	Shame or stigma related to PrEP itself, whether experienced from another person or internalized from a PrEP ad or promotion
Reduce Stigma	The idea that promoting PrEP or discussing PrEP can reduce stigma

Name	Description
Religion & Beliefs	How religion or other spiritual beliefs may affect stigma or shame in some way
Homophobia in religion	Subcode for stigma or shame felt or experienced due to religion, and would include any notion religion or anything like this would be barrier to PrEP promotion
Self-Acceptance	A person accepting of their identity, whether sexual orientation or gender identity and how this may intersect with PrEP promotion or discussion
SO Shame or Stigma	Shame or stigma related to the persons own sexual identity and any notion that seeing persons of same/similar sexual identity in promotional materials might affect (improve or worsen) that stigma or shame
Stigma from ads	The idea that PrEP promotional ads could lead to worsen stigma
Tracked	Subcode for feeling targeted by ads because of a persons identity
Stigma in healthcare	The idea stigma or shame felt by someone at any point while interacting with the healthcare system or health providers
Stigmatizing Risk	The idea that being “at-risk” is stigmatizing in and of itself.
PrEP PROMOTION	Characteristics or aspects of the PrEP promotion itself
Accessible message	Text referring to content or messaging that is easily understandable and approachable
Activity in ad	Referencing persons in ads being active of some kind, such as dancing, hiking, or any other kind of dynamic action

Name	Description
Annoying Ads	Promotions that are disruptive to content or the activity. Also can be used for promotions that are irritating to the person viewing.
App Features	The idea that using an app will ask or promote or in some way trigger a PrEP promotion
Billboards and Signage	Any mention of physical or digital promotion or advertisement using billboards or other types of signs, regardless of posting. May be content that they’ve seen or would like to see
Bystander	Promotion via a person who is present but not actively promoting, perhaps wearing a t-shrt or a sign, etc.
Call to Action	Promotions that feature a motivation or positive behavior change component
Commercial	Any mention of a short video or audio clip embedded to promote PrEP (within, before, or after other content), whether made by a drug company, health authority, or health provider or clinic. May be content that they’ve seen or would like to see.
concise and clear	In Vivo code for a promotion or ad that should be brief and clearly describe some aspect of PrEP
Conversation Starter	The idea that some aspect of the promotion or ad will be so appealing, interesting to initiate or start a dialogue or want to start a dialogue about it
Digital Promotion	Any kind of PrEP promotion or ad that uses a digital platform, exclusive of any print, and is accessed or transmitted via digital technology
Discreet Promotion	Any text referring to promotion that is discreet, which may be in private, 1:1, or intimate setting.
Drag Shows	Drag shows of any variety as a platform to promote or advertise PrEP as a product or a service. May be content that they’ve seen or would like to see.

Name	Description
Events	PrEP promotion at events or gatherings for activities, such as sporting events, balls, drag shows, Pride festivals, etc.
Eye Catching	Mentions any idea about a promotion or ad being visually appealin, particularly if it causes further evaluation or inspection of the promotion or ad.
Graphic	Non-video content, such as a picture or a graphic. Examples might be photos, pictures, figures, graphics, charts, etc.
Humorous	Promotions that feature a humorous or comedic tone
Hypersexualized	In vivo code for PrEP ads that contain some aspect or degree of sexual explicitness
Identifying PrEP Barriers	Promotions or ads that identify and mitigate common barriers to PrEP, such as access, location, cost. This is not for ads about informing about PrEP effectiveness, safety, etc.
Influencer	A notable person or celebrity who may promote or discuss various things.
Music in ad	Any mention of music associated with PrEP promotion or ads
'not forceful'	In vivo code for passive promotion or promoting without “sales pitch”
Outreach	Notion that PrEP can be promoted through outreach events or activities
Personal Experience	Any mention of including a person’s experience (anecdotes, stories, etc.) with accessing or using PrEP
Print Materials	Any mention of physical (non-digital) PrEP promotional materials or ads, including flyers, post cards, mailers, etc.

Name	Description
Promotion by Clinics	Mentioning clinics or providers promoting PrEP or informing about PrEP. This does not include promotion by drug companies or health authority institutions
Radio	Any mention of using radio (whether airwave or steaming) to promote PrEP
School	School as a venue for passive (posting signage) or active (lessons, instruction) about any aspect of PrEP
This is low budget, y'all. Let's do better	In vivo code for perceived low-effort or poorly made PrEP promotions or ads that do not promote PrEP well or may even detract from promoting PrEP
Trivial or little details too much	Promotions that contain too much information or details that are overwhelming to the viewer
TV	Any text referring to PrEP promotion that is viewed by broadcast, cable, or satellite TV. This excludes streaming services.
Venues	Any mention of promotion through a venue or physical establishment, not otherwise mentioned as a code (i.e., school, clinic)
Video	Any mention of using video to promote any aspect of PrEP. This can include streaming, TV, commercials. Anything that is non-print and non-static. This code is for the idea of video as format, rather than the placement or distribution of the video itself.
